# Matrix metalloproteinases 2 and 9 increase permeability of sheep pleura in vitro

**DOI:** 10.1186/1472-6793-12-2

**Published:** 2012-03-16

**Authors:** Eleni Apostolidou, Efrosyni Paraskeva, Konstantinos Gourgoulianis, Paschalis-Adam Molyvdas, Chrissi Hatzoglou

**Affiliations:** 1 Department of Physiology, University of Thessaly Medical School, Larissa, Biopolis 41110, Greece; 2 Department of Respiratory Medicine, University of Thessaly Medical School, University Hospital of Larissa, Larissa, Biopolis 41110, Greece

## Abstract

**Background:**

Matrix metalloproteinases (MMPs) 2 and 9 are two gelatinase members which have been found elevated in exudative pleural effusions. In endothelial cells these MMPs increase paracellular permeability via the disruption of tight junction (TJ) proteins occludin and claudin. In the present study it was investigated if MMP2 and MMP9 alter permeability properties of the pleura tissue by degradation of TJ proteins in pleural mesothelium.

**Results:**

In the present study the transmesothelial resistance (R_TM_) of sheep pleura tissue was recorded in Ussing chambers after the addition of MMP2 or MMP9. Both enzymes reduced RTM of the pleura, implying an increase in pleural permeability. The localization and expression of TJ proteins, occludin and claudin-1, were assessed after incubation with MMPs by indirect immunofluorescence and western blot analysis. Our results revealed that incubation with MMPs did not alter neither proteins localization at cell periphery nor their expression.

**Conclusions:**

MMP2 and MMP9 increase the permeability of sheep pleura and this finding suggests a role for MMPs in pleural fluid formation. Tight junction proteins remain intact after incubation with MMPs, contrary to previous studies which have shown TJ degradation by MMPs. Probably MMP2 and MMP9 augment pleural permeability via other mechanisms.

## Background

Matrix metalloproteinases (MMPs) 2 and 9 are two gelatinase members which have been measured and found to be elevated in pleural exudates of different origin (parapneumonic, malignant, tuberculous). MMPs consist a family of proteolytic enzymes that break down virtually all the protein components of the extracellular matrix. The balance between matrix deposition and degradation is tightly regulated in human tissues and a disruption of this balance has been implicated in several pathological conditions such as cancer, cardiovascular diseases and arthritis [1,2]. MMP2 and MMP9 are thought to be involved in pleural fluid accumulation in the pleural cavity. The disruption of the integrity of the mesothelial layer or the underlying basement membrane, and therefore the facilitation of fluid influx into the pleural space has been proposed as a possible mechanism [3], although no study has been so far conducted.

MMPs have been correlated with the induction of increased capillary permeability in several inflammatory conditions, such as brain and myocardium ischemia injury and diabetic retinopathy [4-6]. In in vitro studies of endothelial cells, MMPs increase paracellular permeability by disrupting tight junction barrier [5,7,8]. Tight junctions (TJs) are a specific type of cell-cell contacts that obstruct paracellular pathway for solute diffusion and they regulate the paracellular passage of small molecules such as water and ions [9]. The two major constituent proteins of TJs are occludin and claudin and the disruption of these proteins in several culture systems has been correlated with increased water and solute flux [9].

This study was designed in order to investigate if MMP2 and MMP9 increase the permeability of sheep pleura and thus contribute to the pathogenesis of pleural effusion formation. The effect of MMP2 and MMP9 on TJ proteins occludin and claudin-1 was examined in order to investigate if MMPs alter paracellular permeability of the mesothelial layer.

## Results

### MMP2 and MMP9 decrease the transmesothelial resistance of parietal and visceral sheep pleura

**Figure 1:**
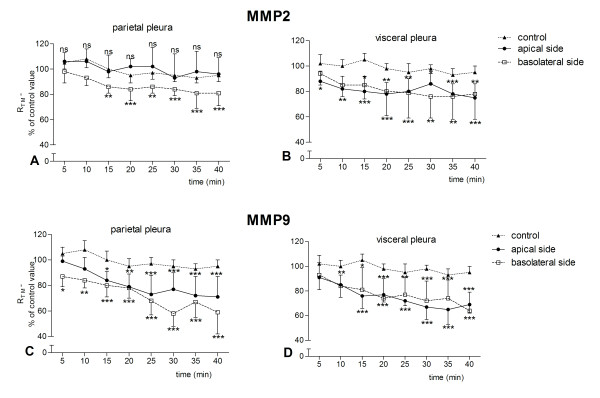
**Effect of 0.1 ng/ml MMP2 and MMP9 on the transmesothelial resistance (R_TM_) of sheep pleura**. A time-dependent decrease in transmesothelial resistance (R_TM_) occurred at both parietal and visceral pleura and on both the apical and basolateral side. For MMP2 the decrease on the apical side of parietal pleura is not significant. Data are given as mean ± SD (n = 6). ns: non-significant, * *p *< 0.05, ** *p *< 0.001 and *** *p *< 0.0001 compared to control R_TM _(ANOVA test with Dunett post-test).

**Figure 2:**
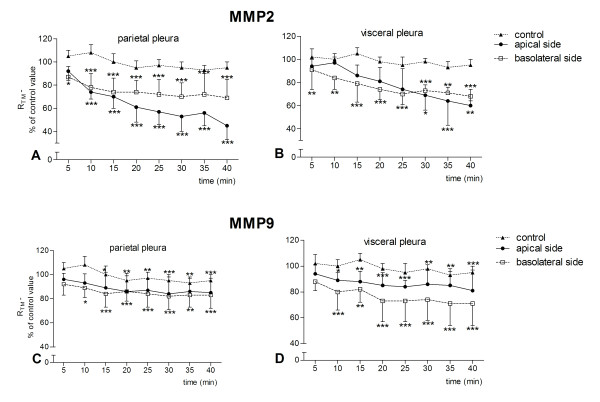
**Effect of 20 ng/ml MMP2 and MMP9 on the transmesothelial resistance (R_TM_) of sheep pleura**. A time-dependent decrease in transmesothelial resistance (R_TM_) of sheep pleura occurred after the addition of MMP2 or MMP9 at concentration 20 ng/ml. The time-dependent response occurred at both parietal and visceral pleura and on both the apical and basolateral side. Data are given as mean ± SD (n = 6). ns: non-significant, * *p *< 0.05, ** *p *< 0.001 and *** *p *< 0.0001 compared to control R_TM _(ANOVA test with Dunett post-test).

We treated parietal and visceral sheep pleura specimens with increasing doses of MMP2 and MMP9 (0.1, 1, 10 and 20 ng/ml) and the R_TM _was measured over a 40-min period. The R_TM _decreased in all concentrations studied and this decrease occurred at both parietal and visceral pleura and on both the apical and basolateral side. The decline in R_TM _suggests for an increase in the permeability of the tissue. As control R_TM _, we regarded the R_TM _of the tissue before MMP was added. The mean R_TM _of parietal pleura was calculated to be 15 ± 3 Ωcm^2 ^and that of visceral pleura 17 ± 4 Ωcm^2^. Sheets of pleura specimens, at which no MMPs where applied, showed a stable value of R_TM _at least for a period of 40 min (Figures 1 and 2, control experiments).

A time-dependent response occurred both apically and basolaterally for all four concentrations studied. After the addition of MMPs, the R_TM _decreased significantly and declined progressively thereafter up to the 40 min of incubation when the experiment was terminated (Figures 1 and 2). A significant drop in resistance occurred within 5-15 min for both concentrations 0.1 (Figure 1) and 20 ng/ml (Figure 2). However a delayed response was observed when 0.1 ng/ml MMP2 was incubated with the apical side of the visceral membrane (Figure 2b). The effect of 0.1 ng/ml MMP2 on the apical side of the parietal pleura was not significant (Figure 1a). The changes in R_TM _for concentrations 1 and 10 ng/ml were comparable to these for 0.1 and 20 ng/ml (data not shown).

**Figure 3:**
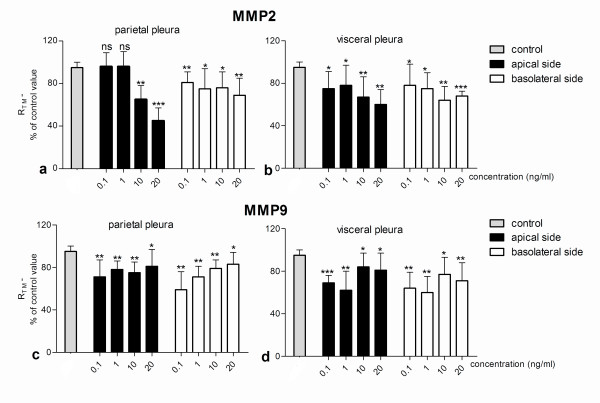
**Dose-dependent decrease in transmesothelial resistance (R_TM_) of sheep pleura after the addition of MMP2 or MMP9**. Different concentrations (0.1, 1, 10 and 20 ng/ml) of MMP2 and MMP9 were added at parietal and visceral pleura and on the apical and basolateral side and the R_TM _was measured on the 40 min. The changes in R_TM _are expressed as percentage difference of the control value (100%), which is the R_TM _value just before the addition of MMP. Data are given as mean ± SD (n = 6). ns: non-significant, * *p *< 0.05, ** *p *< 0.001 and *** *p *< 0.0001 compared to control R_TM _(paired t-test).

A dose-dependent response was observed for both MMPs studied (Figure 3): for MMP2 the decrease in R_TM _was greatest on the apical side of parietal pleura at concentration 20 ng/ml (45 ± 12% of the control value). For MMP9 the greatest effect was observed on the basolateral side of the parietal pleura at the low concentration of 0.1 ng/ml (59 ± 17% of the control value).

### TIMP2 partially prevented the decrease in R_TM _induced by MMPs

**Figure 4:**
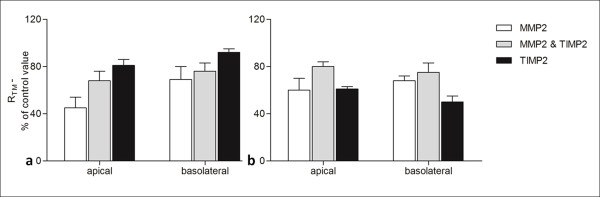
**TIMP2 reverses partially the effect of MMP2 on pleural permeability**. TIMP2 was added at concentration 200 ng/ml at on the apical and basolateral side of parietal(a) and visceral(b) pleura. MMP2 was added at the same time at concentration 20 ng/ml. TIMP2 seems to prevent partially the decline in R_TM _induced by MMP2 on the 40^th ^minute of the experimental procedure. Data are given as mean ± SD. n = 6 for MMP2, n = 3 for combined MMP2 and TIMP2, n = 3 for TIMP2.

**Figure 5:**
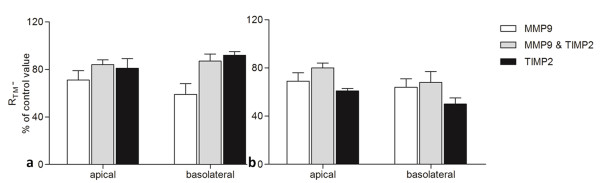
**TIMP2 reverses partially the effect of MMP9 on pleural permeability**. TIMP2 was added at concentration 200 ng/ml on the apical and basolateral side of parietal(a) and visceral pleura(b). MMP9 was added at the same time at concentration 0.1 ng/ml. TIMP2 seems to prevent partially the decline in R_TM _induced by MMP9 on the 40^th ^minute of the experimental procedure. Data are given as mean ± SD. n = 6 for MMP9, n = 3 for combined MMP9 and TIMP2, n = 3 for TIMP2.

Next, the MMP inhibitor TIMP2 (200 ng/ml) was applied to the pleura either alone or simultaneously with MMP2 at concentration 20 ng/ml or MMP9 at concentration 0.1 ng/ml. These concentrations of MMPs were chosen because of their maximum effect on R_TM_. TIMP2 was shown to partially prevent the MMP2 (Figure 4) and MMP9-induced (Figure 5) fall in R_TM_. This effect was obvious on both the parietal and visceral pleura. Moreover, the application of TIMP2 by itself tended to increase pleural permeability, particularly at visceral pleura and at its basolateral side, as the R_TM _was 50 ± 5% of control value on the 40^th ^min (Figure 5d).

### MMP2 or MMP9 do not alter occludin and claudin-1 immunostaining at cell cultures

**Figure 6:**
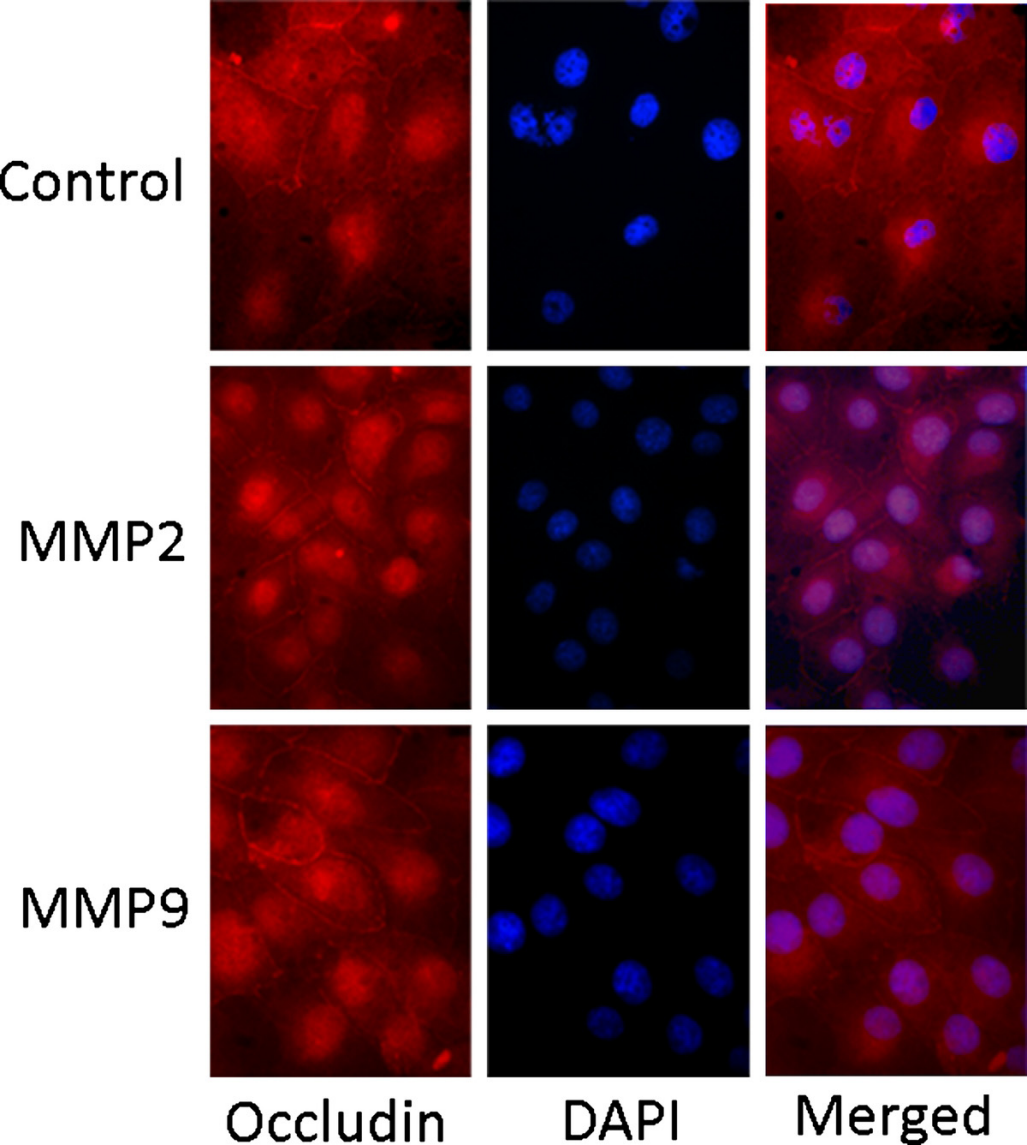
**Immunofluorescent microcopy of pleural mesothelial cells treated or not with MMP2 and MMP9 using an anti-occludin antibody**. Both control cells and cells treated with MMP2 or MMP9 displayed a continuous staining at cell periphery. Nuclei were stained with Dapi.

**Figure 7:**
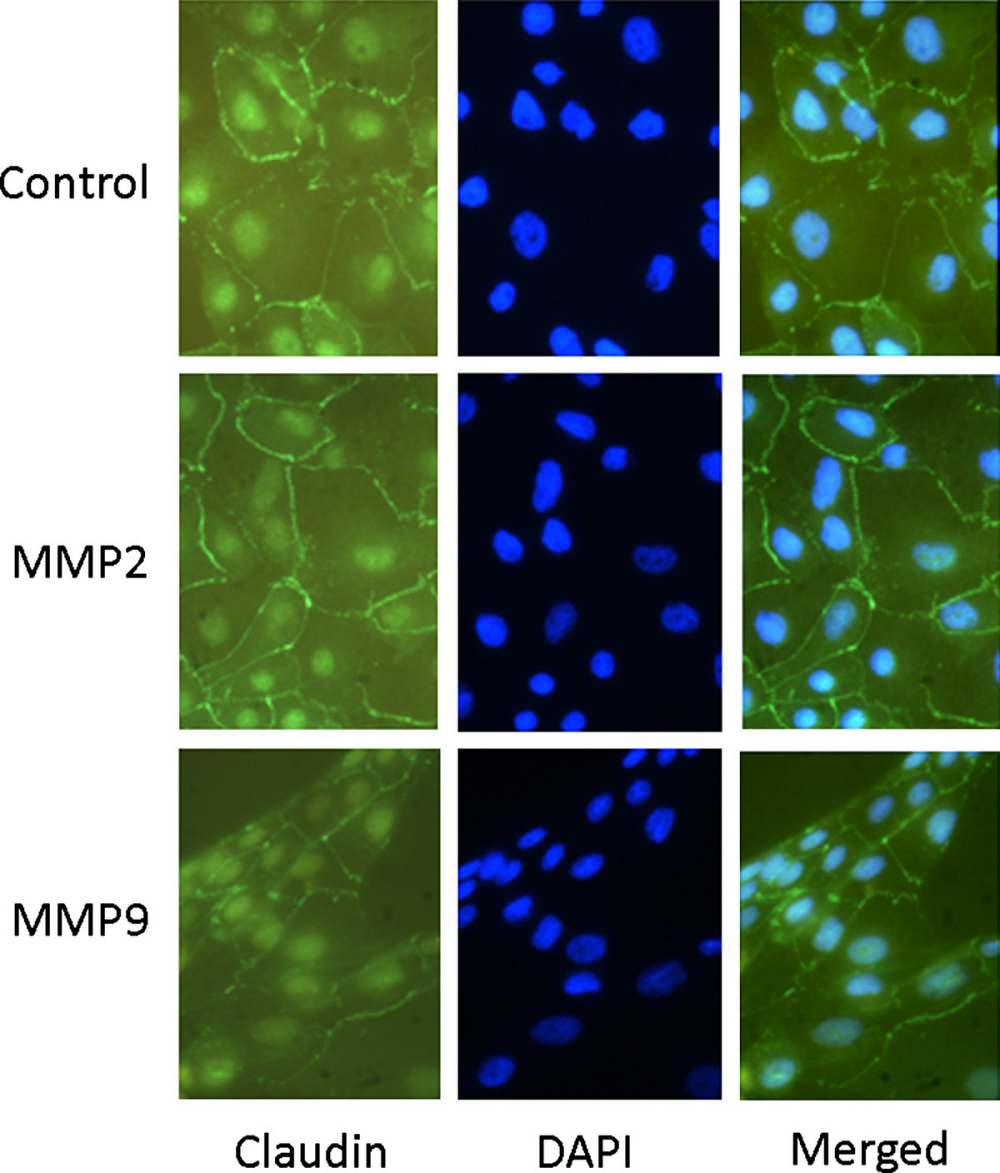
**Immunofluorescent microcopy of pleural mesothelial cells treated or not with MMP2 and MMP9 using an anti-claudin-1 antibody**. Control cells displayed a continuous staining at cell periphery. The same pattern occurred also for cells treated with MMP2 or MMP9. Nuclei were stained with Dapi.

We next investigated if the increase in transmesothelial permeability, as occurred by Ussing chamber experiments, is accompanied by loss of tight junction proteins by indirect immunofluorescence. The mesothelial cells in control experiments showed a clear membrane pattern for occludin (Figure 6) and claudin-1 staining (Figure 7). The staining was continuous and decorated the cell periphery. Because cells were well grown to confluency and occludin and claudin-1 are located at sites of lateral membrane with cell-cell contact, a continuous line resembling to "honeycomb" pattern was obvious. Non-specific staining of the nucleus occurred. The incubation of mesothelial cells with MMPs had no effect on occludin and claudin-1 immunostaining (Figures 6 and 7, respectively), implying that MMPs do not alter occludin and claudin-1 localization at tight junctions.

### MMP2 or MMP9 do not alter occludin and claudin-1 expression at mesothelial cells

**Figure 8:**
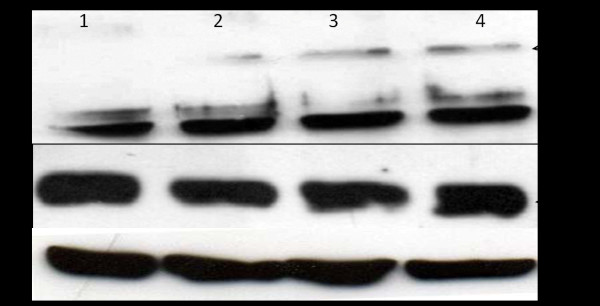
**Immunoblot analysis of TJ proteins after treatment with MMPs**. Occludin appears as a 65 KDa protein **(a) **and claudin-1 migrates as a 28 KDa band **(b)**. lane 1 = control, no incubation, lane 2 = control, incubation for 1 h at 37°C, lane 3 = incubation with MMP2 for 1 h at 37°C, lane 4 = incubation with MMP9 for 1 h at 37°C. Equal amounts of protein were loaded to each lane (15 μg protein/lane). Antibody against β-actin was used to control the equal amounts of proteins in the lysates **(c)**. No alteration to the tight junction proteins occludin and claudin-1 was observed after incubation with MMPs.

Western blotting of mesothelial cell extracts showed no alteration for occludin and claudin-1 staining (Figure 8). Occludin migrated as two main bands with apparent molecular weights from 60 to 65 KDa. An additional band at about 85 KDa was also detected (Figure 8a). Claudin-1 was detected as a 28 kDa band (Figure 6b). Neither loss of band intensity nor the appearance of new protein fragments was observed. These findings imply that MMP2 and MMP9 do not degrade TJ proteins.

## Discussion and Conclusions

In the present study MMP2 and MMP9 decreased the transmesothelial resistance of parietal and visceral sheep pleura in in vitro experiments. This decrease supports that MMPs augment pleural mesothelial permeability. The paracellular pathway, through which mesothelial permeability may increase, was studied. After incubation of primary mesothelial cells with MMP2 or MMP9, mesothelial monolayer integrity was not disrupted and the expression of TJ proteins occludin and claudin-1 in the cytoplasmic membrane remained intact.

MMP2 and MMP9 were selected for our study because they have been found elevated in exudative pleural effusions of different origin [3,10,11]. MMP2 is expressed and secreted constitutively in the pleural cavity by mesothelial cells [12]. In exudates high levels of MMP2 may be due to increased expression as a result of stimulation of mesothelial cells by cytokines or other cells, such as mononuclear cells, might collaborate in MMP2 release [10]. MMPs can degrade almost all components of the extracellular matrix (ECM) and to date it is widely known that MMPs can cleave not only ECM components, such as collagen and elastin, but also non-ECM protein substrates, such as cell surface molecules, ECM-bound growth factors and cytokines released on the ECM [13,14].

In the present study the decrease in transmesothelial electrical resistance of sheep pleura that occurs after incubation with MMP2 or MMP9 suggests for an increase in the permeability of the pleura. As expected for an enzyme, the decrease in resistance of the pleura was time-dependent with the maximum decline in resistance occurring on the 40 min. Regarding the dose-dependent effect, a remarkable difference between MMP2 and MMP9 occurred: for MMP2 the greatest increase in permeability occurred at the highest concentration studied, which is 20 ng/ml. On the contrary, for MMP9 the greatest increase in permeability occurred at the lowest concentration studied, which is 0.1 ng/ml. The reason for this discrepancy is not elucidated but we should take into account differences between enzyme kinetics and substrate specificity.

TIMP2 is a tissue inhibitor of metalloproteinases and inhibits MMPs activity by binding noncovalently to their active site [15]. TIMP2 is found in exudates whereas in transudates its levels are usually no detectable [3]. The application of TIMP2 on the pleura inhibited the decrease in R_TM _which was induced by MMPs on both the parietal and visceral pleura. This finding enhances our previous finding and suggests that the decline in R_TM _is an effect caused by MMPs and not a non-pecific result. TIMP2 was selected at the present study because it is 2-9 times more effective than TIMP1 for the inhibition of MMP2 and MMP9 activity [16]. Moreover, TIMP2 tended to increase pleural permeability particularly at visceral pleura and at its basolateral side, when it was applied alone at the pleura. This finding can be interpreted considering the growth-promoting-activity that has been attributed to TIMP2 [17]. A wide range of human, bovine and mouse cells proliferate when incubated with TIMP2. Indeed, receptors of TIMP2 have been identified at the surface of the above cells [18]. One possible explanation is that TIMP2 acts on mesothelial cells via receptor-binding and influences mesothelial permeability, as is the case for numerous growth factors.

We next looked for a possible mechanism explaining the impact of MMPs on the transmesothelial resistance and asked whether TJ proteins, occludin and claudin-1, may be hydrolyzed by MMPs. TJs are a specific type of cell-cell contact which are located in the most apical region of the lateral plasma membrane. The paracellular passage of small molecules, such as water and solutes, is highly regulated by the TJ proteins, including occludin and claudin-1 [9]. Indirect immunofluorescence experiments for occludin and claudin-1 showed a continuous staining at cell periphery which was not weakened and remained at the cell borders after incubation with MMPs. Similarly, western blot revealed that these proteins are expressed at pleural mesothelium under normal conditions and are not proteolytically disrupted by MMPs. More specifically for occludin, western blot analysis revealed three distinct bands: at 60 KDa, 85 KDa and a broad band at about > 60 KDa. These bands may be due to occurrence of splice variants or post-transational modification, i.e phosphorylation. Previous studies have shown that occludin is widely phosphorylated on serine and threonine resides and modulation of occludin phosphorylation regulates cellular localization and paracellular permeability [19,20]. Some degree of phosphorylation may also be the case in the present study. As far as the 80 KDa band is concerned, we cannot rule out the possibility that this band corresponds to a protein complex between occludin and claudin (MWs 60 and 28 KDa respectively). Occludin and claudin do not interact directly to each other, but they crosslink by integral TJ proteins, such as ZO-1, ZO-2 and ZO-3 [9]. The intensity of the above band increased after MMPs incubation, implying that MMPs interfere with occludin and claudin interactions. Because the 80 KDa band appeared enhanced to all three western blot experiments that were performed, it is less likely to represent an artifact. The mesothelial cells used for immunofluorescence and western blotting derived from upper-, middle- and lower-heighted visceral pleura and no difference between them occurred.

Our data is contradictory to previous results which have revealed MMPs as major contributors to the control of paracellular permeability by proteolytic degradation of TJ proteins. More specifically, MMPs have been correlated with an increase in capillary permeability that follows ischemia-reperfusion injury in brain [8], in myocardium [4], in lung [21] and in kidney [22]. These studies attributed to MMPs an elementary role in the inflammatory process and the breakdown of the paracellular capillary permeability during inflammation. In some studies a selective cleavage of TJ proteins occurred [7,23] but the results of our study clearly showed that TJ integrity is not disrupted at mesothelial cells by MMPs. It is possible that other substrates, different from occludin and claudin-1, are a molecular target for MMPs at pleural mesothelium. For example, MMPs have been found to disrupt or reorganize the basement membrane of endothelial cells and thus result to increased permeability [24,25]. Moreover, adherent junctions are also degraded by MMPs and their hydrolysis leads to TJ disassembly and to increased permeability [22,26].

The limitations of the present study are the followings: Firstly, the Ussing chamber technique investigates permeability alterations provoked on mesothelial membrane, which consists a confluent membrane between the apical and basolateral compartment of a chamber. However, under in vivo conditions it is possible that MMPs act not only on mesothelial cells but also on vascular capillaries lying beneath the basement membrane. Secondly, the precise role of the mesothelial layer at pleural fluid turnover is not fully established. Although we used to believe that mesothelial cells are leaky and display no resistance at pleural fluid passage, more recent investigations indicate that the permeability to solutes of mesothelium is of the same order of magnitude as that of the capillary endothelium [27,28]. This means that pleural fluid is a filtrate of pleural capillaries and mesothelium too. Moreover, damage of pleural mesothelial monolayer by lipopolysaccharide (LPS), thrombin and bacteria increase pleural permeability to proteins and demonstrate to play a central role in the formation of effusions [29,30]. Finally, sheep pleura resembles to human pleura as far as morphology and function is concerned. On both pleurae the blood supply comes from the systemic circulation and microscopically two different types of mesothelial cells are found: the cyboidal cells with less developed TJs and flattened cells with more TJs [31]. However, further studies should be performed at human pleura in order to confirm the results of the present study.

Our findings imply an important role for MMPs in the pathogenesis of pleural effusions. Under normal conditions, MMPs and especially MMP2 are found only in small amounts in the pleural fluid. The balance of MMPs in pleural fluid may serve the degradation and turnover of ECM that underlies mesothelial cells and which normally occurs in low rates. However in pleural exudates MMP2 and MMP9 levels increase, as shown from previous studies, and a role for MMP2 and MMP9 in pleural fluid formation is proposed by our study. Tight junctions do not apparently loosen in mesothelial cells after the addition of MMPs. It is possible that other mechanisms exist through which MMPs increase mesothelial permeability. The revelation of the mechanims by which MMPs compromise the mesothelial barrier will provide a better understanding of the pathogenesis of pleural fluid formation.

## Methods

### Specimen collection and preparation of sheep pleura

Intact sheets of visceral and parietal sheep pleura were obtained from adult female sheep. The samples were collected from the slaughterhouse immediately after the death of the animals (time of warm ischemia close to 0 minutes). Pieces of parietal pleura were carefully stripped from the chest wall whereas those of visceral pleura were carefully stripped from the underlying lung. Parietal and visceral pleura were examined for evidence of holes or adherent tissue and were discarded if they were not intact. Immediately after removal, the pleural tissue from the animals was placed in oxygenated Krebs-Ringer bicarbonate (KRB) solution at 4°C and transferred to the laboratory within 30 minutes. The KRB solution was balanced at pH 7.4 and bubbled with 95%O_2_-5%CO_2_. The solution contained (in mM) 117.5 NaCl, 1.15 NaH_2_PO_4_, 24.99 NaHCO_3_, 5.65 KCl, 1.18MgSO_4_, 2.52 CaCl_2_, and 5.55 glucose.

### Electrophysiological transmesothelial measurements

The effect of MMPs on pleural permeability was studied by conducting Ussing experiments under open circuit conditions. The pleura was mounted carefully in Ussing chambers (K Mussler Scientific Instruments, Aachen, Germany) with an opening surface area of 1 cm^2^. Tissues were bathed with 4 ml KRB solution on each side of the membrane and were continuously oxygenated with 95%O_2_-5%CO_2_. Two pairs of Ag/AgCl electrodes monitored the transmesothelial potential difference (Pd, in mV) and the transmesothelial resistance (R_TM_, in Ωullet cm^2^) under open circuit conditions.

Transmesothelial electrical parameters were measured before and after the addition of active MMP2 or MMP9 (Calbiochem, San Diego, California, USA) at four different concentrations 0.1, 1, 10 and 20 ng/ml. In some experiments TIMP2, a tissue inhibitor of metalloproteinases, was added at concentration 200 ng/ml. MMPs and TIMP2 were added both apically and basolaterally and the electrical parameters were monitored over a period of 40 minutes (at minutes 5, 10, 15, 20, 25, 30, 35, 40). After the addition of the above substances, alterations in the R_TM _were expressed as the change from the starting value. Activity of matrix metalloproteinases is temperature-dependent, therefore measurements of transmesothelial electrical parameters were conducted at 37°C. The mesothelial cell membrane facing the fluid side is here called the apical membrane, and that facing the blood side is called the basolateral membrane. The voltage response to applied current pulses of 50 μA amplitude and 200 msec duration was measured. The transmesothelial resistance was calculated deducting automatically the resistance of the solution.

### Cell cultures

Primary cultures of sheep pleural mesothelial cells were prepared (modified from Stylianou et al [32]). Briefly, specimens of intact visceral sheep pleura were obtained from the slaughterhouse immediately after the death of the animal. The procedure was performed under sterile conditions and pieces of approximately 6 cm^2 ^were placed in M199 media (Invitrogen, Carlsbad, USA) supplemented with 10% fetal bovine serum (FBS), 100 U/ml penicillin and 100 μg/ml streptomycin. The specimen was transferred on ice to the laboratory, washed with PBS and subjected to enzymatic disaggregation. The specimen was in this incubated for twenty minutes at 37°C in disaggregation solution that contained 0.125% trypsin, 0.01% EDTA and 0.1% glucose in PBS solution. After incubation the pleura membrane was discarded and the suspension was centrifuged at 100 × g for five minutes at 4°C. The resultant supernatant was discarded and the cell pellet was suspended in 10 ml prewarmed M199 media supplemented with 10% fetal bovine serum (FBS), 100 U/ml penicillin, 100 μg/ml streptomycin, 0.4 μg/ml hydrocortisone, 10 mg/ml insulin, 50 mg/ml transferin and 200 mM L-glutamine and subjected to recentrifugation at 100 × g for five minutes at 4°C. Finally the supernatant was discarded, the cell pellet was resuspended in 5 ml of the same medium and seeded in 25 cm^2 ^tissue culture flasks. The medium was changed every third or fourth day. Cultures contaminated with spindle-like cells, representing probably fibroblasts, were discarded. Confluency of the cells was regarded as a prerequisite for their experimental use. Passages 1 and 2 were used for immunofluorescence experiments and western blot.

### Indirect immunofluorescence

For immunofluorescence experiments, mesothelial cells were grown on fibronectin-coated glass coveslips and confluency was reached about 7 days later. After two subsequent washes with PBS, cells were incubated in serum-free media with MMP2 or MMP9 at concentration 10 μg/ml for one hour at 37°C and then were fixed with 3% formaldehyde for 5 minutes at room temperature. Control cells received no MMP treatment and were incubated with serum-free media. After washing, the cells were permeabilized with 1% Triton X-100 in PBS for 15 minutes at 4°C. Samples were washed with PBS and soaked in blocking solution, which consisted of 3% BSA and 0.1% Tween-20 in PBS for 16 hours. Coverslips were incubated with polyclonal anti-occludin (dilution 1:50; Zymed Laboratory, San Fransisco, USA) or polyclinal anti-claudin-1 antibody (dilution 1:50; Zymed Laboratory, San Fransisco, USA) in PBS containing 1% BSA/0.1% Tween-20 for 1 hour at room temperature. Cells were rinsed three times in 1% BSA/0.1% Tween-20/PBS and incubated with a FITC- or CY3-conjugated anti-rabbit IgG secondary antibody (dilution 1:50), for occludin and claudin repectively, for 30 minutes at room temperature. Finally coverslips were washed and mounted with Vectashield containing Dapi. Images were collected with a Leica DFC480 camera (LAS software version V2.3.1R1) on an Axioscope 40 Zeiss microscope.

### Western blot

Mesothelial cells were grown in culture dishes and cellular extract was obtained as described previously by Giebel et al [5]. Briefly, mesothelial cells were washed with cold PBS and cellular extract was obtained after scraping the culture dish with 200 μl lysis buffer (0.1% Triton X-100 in 100 Mm PO_4 _buffer). The cellular extract was incubated for 30 min on ice and then was subjected to centrifugation at 100 × g for 30 min at 4°C. The pellet was discarded and the supernatant was divided into four equal aliquots each of which had a volume 20 μl. The protein concentration was measured by Bradford method (Bio rad) and each aliquot contained 15 μg proteins. Two aliquots were used as controls and one of them was incubated at 37°C for one hour. Additional aliquots received either MMP2 or MMP9 at concentration 5 μg/ml and were incubated at 37°C for one hour. At the end of incubation period, SDS sample buffer was added to all aliquots and incubation at 95°C for 3 min followed. Samples were loaded on 10% polyacrylamide gel for electrophoresis and then proteins were transferred to a nitrocellulose membrane. The quality of the transfer was controlled by Ponceau staining of the membrane. The membrane was blocked in 5% milk in PBS-Tween-20 for 30 min at room temperature and then incubated with primary antibodies (polyclonal anti-occludin and anti-claudin-1 at dilution 1:250) for 16 hours at 4°C. After 3 washes with PBS-Tween-20, the membrane was incubated for 1 hour at room temperature with HRP-secondary antibody (anti-rabbit IgG at dilution 1:3000) and the immunoreactive bands were detected with enhanced chemiluminescence (Roche).

### Statistical analysis

Statistical analysis for quantative experiments was performed using GraphPad Prism 5. For permeability experiments, comparisons between control and MMP-treated tissues were made using one way ANOVA test with Dunnett post-test. For short-circuit experiments, the comparison between control experiments and experiments with application of MMPs was performed with unpaired t-test. Values of p < 0.05 were regarded as significant.

## Abbreviations

MMPs: Matrix metalloproteinases; TJs: Tight junctions; R_TM_: Transmesothelial resistance; SD: Standard deviation; TIMP2: Tissue inhibitor of metalloproteinases 2; ECM: Extracellular matrix; FBS: Fetal bovine serum.

## Authors' contributions

EA wrote this manuscript. EP contributed to the performance of indirect immunofluorescence and western blotting. CH, KG and PAM conceived the study and participated in the design of the experiments. All authors read and approved the final manuscript.

## References

[B1] Kerrigan JJ Kerrigan JJ Mansell JP Sandy JR Matrix turnover J Orthod 2000 27 227 233 11099555 10.1179/ortho.27.3.227

[B2] Nagase H Visse R Murphy G Structure and function of matrix metalloproteinases and timps Cardiovasc Res 2006 69 562 573 10.1016/j.cardiores.2005.12.002 16405877

[B3] Eickelberg O Sommerfeld CO Wyser C Tamm M Reichenberger F Bardin PG Soler M Roth M Perruchoud AP MMP and TIMP expression pattern in pleural effusions of different origins Am J Respir Crit Care Med 1997 156 1987 1992 9412584 10.1164/ajrccm.156.6.9704112

[B4] Danielsen CC Wiggers H Andersen HR Increased amounts of collagenase and gelatinase in porcine myocardium following ischemia and reperfusion J Mol Cell Cardiol 1998 30 1431 1442 10.1006/jmcc.1998.0711 9710810

[B5] Giebel SJ Menicucci G McGuire PG Das A Matrix metalloproteinases in early diabetic retinopathy and their role in alteration of the blood-retinal barrier Lab Invest 2005 85 597 607 10.1038/labinvest.3700251 15711567

[B6] Rosenberg GA Estrada EY Dencoff JE Matrix metalloproteinases and TIMPs are associated with blood-brain barrier opening after reperfusion in rat brain Stroke 1998 29 2189 2195 10.1161/01.STR.29.10.2189 9756602

[B7] Lohmann C Krischke M Wegener J Galla HJ Tyrosine phosphatase inhibition induces loss of blood-brain barrier integrity by matrix metalloproteinase-dependent and -independent pathways Brain Res 2004 995 184 196 10.1016/j.brainres.2003.10.002 14672808

[B8] Yang Y Estrada EY Thompson JF Liu W Rosenberg GA Matrix metalloproteinase-mediated disruption of tight junction proteins in cerebral vessels is reversed by synthetic matrix metalloproteinase inhibitor in focal ischemia in rat J Cereb Blood Flow Metab 2007 27 697 709 16850029 10.1038/sj.jcbfm.9600375

[B9] Schneeberger EE Lynch RD The tight junction: a multifunctional complex Am J Physiol Cell Physiol 2004 286 C1213 C1228 10.1152/ajpcell.00558.2003 15151915

[B10] Iglesias D Alegre J Aleman C Ruiz E Soriano T Armadans LI Segura RM Angles A Monasterio J de Sevilla TF Metalloproteinases and tissue inhibitors of metalloproteinases in exudative pleural effusions Eur Respir J 2005 25 104 109 10.1183/09031936.04.00010504 15640330

[B11] Vatansever S Gelisgen R Uzun H Yurt S Kosar F Potential role of matrix metalloproteinase-2,-9 and tissue inhibitors of metalloproteinase-1,-2 in exudative pleural effusions Clin Invest Med 2009 32 E293 E300 19640333 10.25011/cim.v32i4.6621

[B12] Marshall BC Santana A Xu QP Petersen MJ Campbell EJ Hoidal JR Welgus HG Metalloproteinases and tissue inhibitor of metalloproteinases in mesothelial cells. Cellular differentiation influences expression J Clin Invest 1993 91 1792 1799 10.1172/JCI116390 8386195 PMC288160

[B13] Arribas J Coodly L Vollmer P Kishimoto TK Rose-John S Massague J Diverse cell surface protein ectodomains are shed by a system sensitive to metalloprotease inhibitors J Biol Chem 1996 271 11376 11382 10.1074/jbc.271.19.11376 8626692

[B14] McQuibban GA Gong JH Tam EM McCulloch CA Clark-Lewis I Overall CM Inflammation dampened by gelatinase A cleavage of monocyte chemoattractant protein-3 Science 2000 289 1202 1206 10.1126/science.289.5482.1202 10947989

[B15] Woessner F Jr Matrix metalloproteinases and their inhibitors in connective tissue remodeling Faseb 1991 5 2145 2154 1850705

[B16] Kahari VM Saarialho-Kere U Matrix metalloproteinases and their inhibitorsin tumour growth and invasion Ann Med 1999 31 34 45 10.3109/07853899909019260 10219712

[B17] Hayakawa T Tissue inhibitors of metalloproteinases and their cell growth- promoting activity Cell Struct Funct 1994 19 109 114 10.1247/csf.19.109 7954870

[B18] Hayakawa T Yamashita K Ohuchi E Shinagawa A Cell growth-promoting activity of tissue inhibitor of metalloproteinases-2 (TIMP-2) J Cell Sci 1994 107 2373 2379 7844157 10.1242/jcs.107.9.2373

[B19] Sakakibara A Furuse M Saitou M Ando-Akatsuka Y Tsukita S Possible involvement of phosphorylation of occludin in tight junction formation J Cell Biol 1997 137 1393 1401 10.1083/jcb.137.6.1393 9182670 PMC2132539

[B20] Harhaj NS Antonetti DA Regulation of tight junctions and loss of barrier function in pathophysiology Int J Biochem Cell Biol 2004 36 1206 1237 10.1016/j.biocel.2003.08.007 15109567

[B21] Yano M Omoto Y Yamakawa Y Nakashima Y Kiriyama M Saito Y Fujii Y Increased matrix metalloproteinase 9 activity and mRNA expression in lung ischemia-reperfusion injury J Heart Lung Transplant 2001 20 679 686 10.1016/S1053-2498(01)00250-9 11404174

[B22] Covington MD Burghardt RC Parrish AR Ischemia-induced cleavage of cadherins in NRK cells requires MT1-MMP (MMP-14) Am J Physiol Renal Physiol 2006 290 F43 F51 16077081 10.1152/ajprenal.00179.2005

[B23] Chen F Ohashi N Li W Eckman C Nguyen JH Disruptions of occludin and claudin-5 in brain endothelial cells in vitro and in brains of mice with acute liver failure Hepatology 2009 50 1914 1923 10.1002/hep.23203 19821483 PMC2925168

[B24] Fukuda S Fini CA Mabuchi T Koziol JA Eggleston LL Jr del Zoppo GJ Focal cerebral ischemia reduces active proteases that degrade microvascular matrix Stroke 2004 35 998 1004 10.1161/01.STR.0000119383.76447.05 15001799 PMC2979008

[B25] Lacherade JC Van De Louw A Planus E Escudier E D' Ortho MP Lafuma C Harf A Delclaux C Evaluation of basement membrane degradation during TNF-alpha-induced increase in epithelial permeability Am J Physiol Lung Cell Mol Physiol 2001 281 L134 L143 11404256 10.1152/ajplung.2001.281.1.L134

[B26] Navaratna D McGuire PG Menicucci G Das A Proteolytic degradation of VE-cadherin alters the blood-retinal barrier in diabetes Diabetes 2007 56 2380 2387 10.2337/db06-1694 17536065

[B27] Bodega F Zocchi L Agostoni E Macromolecule transfer through mesothelium and connective tissue J Appl Physiol 2000 89 2165 2173 11090563 10.1152/jappl.2000.89.6.2165

[B28] Agostoni E Bodega F Zocchi L Equivalent radius of paracellular "pores" of the mesothelium J Appl Physiol 1999 87 538 544 10444610 10.1152/jappl.1999.87.2.538

[B29] Kroegel C Antony VB Immunobiology of pleural inflammation: potentialimplications for pathogenesis, diagnosis and therapy Eur Respir J 1997 10 2411 2418 10.1183/09031936.97.10102411 9387973

[B30] Antony VB Immunological mechanisms in pleural disease Eur Respir J 2003 21 539 544 10.1183/09031936.03.00403902 12662014

[B31] Wheeldon EB Mariassy AT McSporran KD The pleura: a combined light microscopic and scanning and transmission electron microscopic study in the sheep. II. Response to injury Exp Lung Res 1983 5 125 140 10.3109/01902148309061509 6628347

[B32] Stylianou E Jenner LA Davies M Coles GA Williams JD Isolation, culture and characterization of human peritoneal mesothelial cells Kidney Int 1990 37 1563 1570 10.1038/ki.1990.150 2362409

